# Otoprotective effect of the use of antioxidants on noise exposure in experimental studies with rodents – A systematic review with meta-analysis

**DOI:** 10.1016/j.bjorl.2025.101696

**Published:** 2025-09-26

**Authors:** Gabriela Guenther Ribeiro Novanta, Ana Carolina Odorizzi Zica, Maria Luiza Queiroz Sampaio, Camila de Castro Corrêa, Lucieny Silva Martins Serra, Andre Luiz Lopes Sampaio

**Affiliations:** a Universidade de Brasília, Faculdade de Medicina, Laboratório de Pesquisa em Otorrinolaringologia, Campus Darcy Ribeiro, Asa Norte, DF, Brazil; b Universidade Estadual Paulista (UNESP), Faculdade de Medicina, Campus de Botucatu, Botucatu, SP, Brazil

**Keywords:** Rodentia, Noise, Noise-induced hearing loss, Antioxidants

## Abstract

**Objective:**

To determine the occurrence of otoprotective effect of the use of antioxidants on noise exposure in experimental models with rodents through a systematic review.

**Methods:**

An electronic search was conducted in EMBASE, LILACS, PubMed/Medline, Scopus, Web of Science databases, and in the grey literature of articles published up to June 2022. In the PICO strategy, the studied population consisted of rodent animals exposed to high levels of sound pressure, the intervention consisted of the use of antioxidants, the comparison with the concomitant use of placebo and the results of audiological tests such as Evoked Otoacoustic Emissions and Brainstem Auditory Evoked Potential and the results of histological and immunohistochemical evaluations were collected as outcomes. The analysis of the numerical data was performed according to the Review Manager (Cochrane), using the mean difference as a measure of effect.

**Results:**

35 studies were included in the qualitative synthesis and 27 in the quantitative synthesis. The analysis carried out by CAMARADES indicates that most of the included studies demonstrate methodological quality, and the SYRCLE RoB risk of bias assessment protocol revealed a high risk of bias in all eligible studies. In the analysis of the meta-analysis, the effect generated by the use of antioxidants was notorious in comparison with groups that were exposed to the same noise, without the use of antioxidant. Using the ABR responses at the most commonly used frequencies of 2,000–32,000 Hz as outcomes, it was possible to observe in all analyses an otoprotective effect caused by the use of the antioxidant.

**Conclusion:**

This systematic review with meta-analysis demonstrated the variable otoprotective effect of the use of antioxidants on exposure to sound pressure levels in experimental studies with rodents, despite the high risk of bias and great heterogeneity of the selected studies.

**Level of Evidence:**

1.

## Highlights

• Antioxidants provide protection against the effects of noise.
• Studies that standardize the methodology in noise should be encouraged.
• SYRCLE analysis revealed a high risk of bias in all eligible studies.
• Divergences in methods impair the reliability and replicability.

## Introduction

Noise is a common physical and environmental agent. It is present in various human activities, in domestic background and most of job-related processes, and can be described as a generalized form of pollution.^1^ Noise Induced Hearing Loss (NIHL) is currently one of the most common job-related diseases. According to the National Institute for Occupational Security and Health (NIOSH)^2^ it is estimated that about 22 million U.S. workers are currently exposed to high levels of noise and that all industries present hearing hazards. In addition to the difficulty communicating, these workers may suffer from tinnitus, heart and mental health problems in consequence of the exposure to loud noise.

Cellular and molecular mechanisms related to NIHL have been studied for years and have not been fully elucidated yet. Evidence demonstrates the relationship between noise exposure and the generation of Reactive Oxygen Species (ROS) and excessive Nitric Oxide (NO) synthesis.^3^^,^^4^ With noise exposure, the electron transport chain in the mitochondria uses large amounts of oxygen and eventually produces superoxide and generates higher levels of ROS. Finally, increased ROS generation may occur in cochlear fluids and tissues, damage DNA, and break down lipid and protein molecules in the cell, leading to cochlear damage.^4^, ^5^, ^6^

Rats have been widely used to evaluate morphological, physiological, biochemical and molecular aspects related to noise. Rat’s cochlear structure is comparable to that found in humans and other mammals, with similar transduction mechanisms and synaptic connections, such as rat's cochlear spiral, analogous to what is found in humans. Rat's capability to recover after noise exposure allows the investigation of long-term effects and their models of noise-related injuries provide valuable information for understanding the underlying mechanical changes and identifying therapeutic targets for treatment.^7^

Several substances with antioxidant properties have been studied as a form of protection against neurodegenerative cell death, due to their ability to partially protect cochlear sensory cells in the fight against stress-induced damage,^8^ however, there is still no evidence to support the use of one or more antioxidants as an otoprotective agent.

Studies associate resveratrol with a significant reduction in the expression of Cyclooxygenase-2 (COX-2), which is linked to cytotoxic and neurotoxic damage.^1^ Additionally, NG-nitro-L-Arginine Methyl Ester (L-NAME) has been shown to reduce Nitric Oxide (NO) production in cochlear tissue.^4^ Furthermore, pretreatment with a variety of vitamins A, C, and E has been associated with a reduction in the early formation of free radicals.^8^ However, some studies highlight limitations. Puel et al.^9^ noted that while antioxidants may protect against early cochlear damage, their effectiveness decreases in more advanced hearing loss stages. Furthermore, Schacht et al.^10^ emphasized that while antioxidants show promising experimental models, translating these results to human clinical practice remains challenging due to issues such as bioavailability and dosing.

Due to these small discrepancies found in the literature, the importance of a systematic review study on the subject is highlighted, with the aim of critically evaluating the evidence on antioxidant efficacy in the treatment of cochlear damage and denoting the state of the art of this possible use, pointing out the need for translational studies for the human species. The aim of this study was to evaluate the otoprotective effect of the use of antioxidants on noise exposure in experimental studies, through a systematic review.

## Methods

This is a systematic review of the literature followed by a meta-analysis of the collected data. The search strategy followed the criteria recommended by the guideline Preferred Reporting Items for Systematic Reviews and Meta-Analyses, PRISMA (PAGE, 2021).^11^ The protocol was registered in PROSPERO database by the number CRD42023440346.

The databases used included Medline via Pubmed, Latin American and Caribbean Health Sciences Literature (LILACS) via the Virtual Health Library (VHL), Scopus, Embase, Web of Science, and the grey literature of Google Scholar and Proquest Dissertations & Theses. The search strategies were composed of Medical Subject Headings (MeSH), Health Sciences Descriptors (DeCs), as well as free terms, properly connected by the Boolean operators AND and OR. These terms were adapted for each database using the following keywords in the search strategy, for example: “Rodentia”, “Noise”, “Hearing”, “Hearing Loss”, “Antioxidants”. Regarding the use of grey literature and filtering in Google Scholar used Strict inclusion criteria were applied, such as relevance to the research question, methodological quality, and sufficient data for analysis. Filtering was also based on publication date (limiting to studies published in the last 10 years) and specific keywords highly relevant to the scope of the review.

After the search, references of each database were exported to the EndNote X7.0.1 software, Thomson Reuters, with the record of all duplicated articles found in the scientific literature, and then transported to Rayyan software, for the application of the eligibility criteria. It should be noted that there was no restriction on language or year of publication.

### Eligibility criteria

The PICO strategy (population, intervention, comparison, main outcomes) was used to define the search question and the eligibility criteria of the selected studies. The search question was: Does the use of antioxidant provide an otoprotective effect on rodents exposed to high levels of noise? The population of the selected articles was composed of rodent animals exposed to high levels of sound pressure, the intervention consisted of the use of various antioxidants, the comparison consisted of the concomitant use with placebo or another antioxidant and for the outcomes, the results of audiological tests, such as Evoked Otoacoustic Emissions (EOAE) and Brainstem Auditory Evoked Potential (ABR), and results of histological and immunohistochemical evaluations were used.

Exclusion criteria covered studies with non-rodent animals exposed or not to high sound pressure levels; animals with comorbidities; studies in which the results were not evaluated based on histological and/or immunohistochemical analyses and/or ABR or EOAE tests; studies in which noise was associated with another unhealthy factor (vibration, chemicals); studies conducted with impact noise or artillery, studies in which two or more antioxidants were associated, studies conducted in humans, in vitro, ex vivo and in silico studies; studies of any kind of review; studies with duplicate data; case studies, observational studies; and letter to the editor.

### Study selection

Studies were selected for eligibility in the screening phases considering the inclusion and exclusion criteria. During the first phase, all studies were selected by two independent reviewers based on the analysis of titles and abstracts.

During the second phase, the same reviewers analyzed the full text of each selected article based on the inclusion and exclusion criteria already established and added the exclusion justification for each excluded study. At the end of each selection stage, a consensus meeting was held, and a third reviewer was consulted in cases of divergence. The extracted data were antioxidant, time, intensity and frequency of exposure, and main results and conclusion.

### Risk of study bias

The instruments used to evaluate the analysis quality and the risk of bias were the CAMARADES and the SYRCLE RoBS. Items 4, 6, and 7 of CAMARADES checklist were adapted to this review. The items of both instruments were scored as “yes”, “no” or “not clear”. These adaptations were designed to enhance clarity and transparency, without compromising the integrity of the tool. While it was considered the importance of justifying modifications to maintain validity and reliability, the adjustments made reflect the unique requirements of our study and were carefully considered to maintain the rigor of the original checklist. Moreover, these adaptations were followed by other studies published in the past employing a very similar methodology of the present report.^12^

For analysis, the corresponding percentage of affirmative answers was considered. Therefore, for percentages up to 49%, the risk of bias was considered high, between 50% to 69% the risk was moderate, and above 70% the risk of bias was low.^13^

### Summary of the results

The data extracted for meta-analysis were organized according to common hearing frequencies presented by each study in the ABR assessment. Numerical data analysis was performed and reported according to the Review Manager (Cochrane). The mean difference was used as a measure of effect. A random-effects model was fitted to the data. Heterogeneity (tau²) was estimated using the restricted maximum likelihood estimator (Viechtbauer 2005). In addition to the tau² estimate, the *Q* test for heterogeneity (Cochrane 1954) and the I² statistic were reported. The random-effects model was used for detected outcomes with high heterogeneity (i.e., tau² > 25).

## Results

**Fig. 1:**
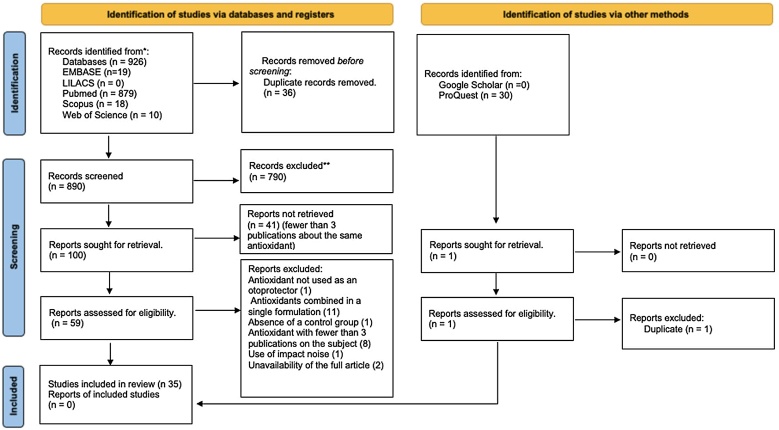
Flowchart of the selection process. From: Page MJ, McKenzie JE, Bossuyt PM, Boutron I, Hoffmann TC, Mulrow CD, et al. The PRISMA 2020 statement: an updated guideline for reporting systematic reviews. BMJ 2021;372:n71. doi: 10.1136/bmj.n71.

In this stage, 926 articles were identified. After removing 36 duplicates, 890 records remained. After the analysis which included the reading of the title and abstract 100 articles were selected. In the second, 59 articles were selected and read in full, of which 35 were included in the study (Fig. 1).

The summary of the articles included for qualitative analysis is described in Table 1.

**Table 1:** Summary of included studies on the otoprotective effect of antioxidant use in noise exposure in experimental studies.

Study	Antioxidant	Time/Intensity/Frequency/Evaluation	Outcomes/ Conclusion
Clifford et al (2011)^10^	D-Met and NAC	6 h / 105 dB / Octave band noise at 4 kHz	DMET group presented significant threshold recovery in the third week, in the frequencies of 4 and 6 kHz p < 0.001 and p < 0.05) and NAC group presented significantly less improvement than DMET group (p < 0.05).
Rewerska, et al (2013)^3^	D-Met	8 h / 110 dB / Octave band noise at 4 kHz	Only the group that received 400 mg/kg showed a significant threshold reduction (p < 0.05).
Samson et al (2008) ^11^	D-Met	4 h / 110 dB / Octave band noise at 4 kHz	On day 14, D-met group showed a significant decrease in threshold shift (p < 0.05) at frequencies of 4 kHz and 8 kHz.
Kil et al (2007)^12^	Ebselen	4 h / 113 dB SPL / 4–16 kHz (octave band)	Better performance was observed in the 14-day treatment group in e ABR thresholds, as well as in the evaluation of OHC loss. In the analysis of estrial area acute changes and GPX1 expression, ebselen-treated rats had a 9.1% increase in the weighted intensity of GPX1 area (p = 0.05) and for acute changes in the spiral ligament, a 9.3% increase of the weighted intensity of GPX1 area (p = 0.05).
Lynch et al (2004)^13^	Ebselen	4 h / 113 dB, 115 dB / 4–16 kHz range.	The dosage of 16 mg/kl provided a significant reduction in permanent threshold shift (p < 0.05 at 3-weeks after noise exposure) and a reduction OHC loss. The cytocochleogram indicated an average loss of 128 ± 43 OHC in rats treated with Ebselen, versus a loss of 339 ± 04 OHC in control animals.
		4 h / 110 dB / 4–16 kHz. Exposure on D0 and after three weeks of the first exposure.	In animals that received two exposures to noise, a significant degree of protection was observed for frequencies of 8 (p < 0.01) and 16 kHz (p < 0.05). Cytocochleogram analysis indicated an average loss of 73 ± 0 OHC in Ebselen-treated rats versus 253 ± 58 OHC in the control group.
Pourbakht, A; Yamasoba, T; (2003)^14^	Ebselen/gavage	5 h / 125 dB SPL / 4 kHz (octave band)	The dose of 30 mg/kl showed more deviations in all frequencies, statistically significant in frequencies of 4, 8 and 16 kHz (p < 0.01). Cytocochleograms showed a reduction in OHC loss and injury width in groups that used ebselen.
Yamasoba et al (2005)^15^	Ebselen – orally	3h / 115 dB SPL / 4 kHz (octave band)	Animals receiving ebselen showed no change in ABR after exposure and the mean area of swollen dendrites in the ebselen-treated animals was significantly lower (p < 0.01) than in the vehicle-treated animals.
Gao et al (2015)^16^	Edaravone	2 h / 110 dB / 2 days / (0.25–4 kHz range, peak at 500 and 1000 Hz	The ABR threshold shift was substantially reduced by edaravone SLNs treatment administered by intratympanic injection, however there was no difference between the groups in the analysis of OHC loss.
Tanaka et al (2005)^17^	Edaravone	3h / 130 Db / 4 kHz centered noise / ABR / Histology	The group that used ederavone 9 -hs after noise exposure had the smallest threshold shifts in ABR (p < 0.01 at the frequencies of 2, 4 and 8 kHz) and the lowest percentage of hair cell loss.
Takemoto et al (2004)^18^	Edaravone	3h / 130 dB / noise centered at 4 kHz.	Threshold shifts at 8 kHz in treated ears were significantly smaller than in control ears (p < 0.05), as was the number of missing/defective outer hair cells after noise exposure.
Chen, et al. (2014)^19^	Hydrogen-saturated saline solution	1 h / 130 dB / narrowband noise centered at 2.5–3.5 kHz	Hydrogen group showed less threshold shift in the ABR and DPOAE amplitude were significantly better compared to the normal saline group. Scanning electron microscopy showed that the hair cells morphology was minimally damaged in animals treated with hydrogen-saturated saline.
Lin, et al. (2011)^20^	Water supersaturated with hydrogen	3h / 115 dB / narrowband noise centered at 4 kHz	In the ABR, on the 14^th^ day after noise exposure, a statistically significant difference was observed between the two groups only at the frequency of 4 kHz (p < 0.05), and in the DPOAE analysis, both groups exhibited almost normal function.
Zhou, et al. (2012)^21^	Hydrogen-rich saline solution	4 h / 115 dB / narrowband noise centered at 4 kHz	In the evaluation carried out on day 7, the group that received a hydrogen-rich solution (HS + NOISE) showed a significant reduction in ABR thresholds (p < 0.01); In DPOAE evaluation, a statistically significant difference was observed in the comparison between HS + NOISE group and NS + NOISE and NOISE ALONE groups (p < 0.01) in most of the analyzed frequencies. The number of missing hair cells was significantly higher in NS + NOISE and NOISEALONE groups than in HS + NOISE and NO TREATMENT groups (p < 0.01).
Diao et al. (2007)^4^	L-NAME	5 h / 115 dB / octave band noise 4 kHz	Group III showed greater threshold shifts at click and at all frequencies (p < 0.05) compared to group IV. A statistically significant difference was observed in the analysis of the mean percentage of OHC loss in the comparison between groups III (loss between 10 and 20%) and IV (5–10%) (p < 0.05). NO level, 3 days after noise exposure, in groups III and IV was 120 ± 35 mmoL/gprot and 75.7 ± 15.3 mmoL/gprot, respectively (statistically significant difference for animals in group III, in relation to groups I and IV (p < 0.001)).
Nagashima et al. (2010)^22^	Tempol and L-NAME	1 h / 110 dB /octave band noise at 8 kHz	Animals that received higher doses of Tempol (3 mg/kl or more) or L-NAME (0.1 mg/kl or more) showed a statistically significant difference in ABR at 4 kHz according to evaluations conducted at D5 (p < 0.05) and D7 (p < 0.01). Tempol (30 mg/kl) and L-NAME (1.0 mg/kl) significantly reduced the increase in the number of positive cells compared to the group exposed to noise for p-SEK-1 (p < 0.05) (p < 0.01), p-JNK (p < 0.01) (p < 0.05) and pc-Jun (p < 0.01) (p < 0.01). There was observed an effect in noise-induced decrease in the level of connexin26 in the lateral wall, comparing the Tempol (30 mg/kl) and L-NAME (1.0 mg/kl) groups with the group exposed to noise, with a statistically significant difference (p < 0.05 and p < 0.01, respectively).
Ohinata et al (2003)^23^	L-NAME / NAC	5 h / 115 dB / 4 kHz octave band noise	Pretreatment with NAC significantly reduced noise-induced threshold shifts at all frequencies (p < 0.05), and attenuated OHC and IHC losses (p < 0.05) compared to the control group. L-NAME was ineffective in protecting thresholds in the ABR and the loss of OHC and ICC. In the NAC group, 8-isoprostane levels were attenuated in the organ of corti, modiolar nucleus and lateral wall. Treatment with L-NAME also reduced 8-isoprostane levels in the modiolar nucleus and lateral wall, but not in the organ of corti.
Ada et al. (2008)^24^	L-NAC	6 h / 110 dB / 1–12 kHz band white noise	NAC group showed less cochlear damage than the group exposed to noise, with fewer cells absent in IHC and OHC. In the analysis by scanning microscopy, NAC group showed stereocilia loss, but with almost normal cell structure and vertical and symmetrical alignment of the stereocilia structures.
Bielefeld et al (2005)^25^	L-NAC	6 h / 4 days / 100 dB / continuous octave band noise centered at 4 kHz	The average of OHC losses for the control group were 29%–41% at the 2–8 kHz range. L-NAC group had an average of OHC loss of less than 10% in the same area (statistically significant difference p < 0.001).
Bielefe et al (2007)^26^	NAC	6 h / 105 dB / continuous octave band noise centered at 4 kHz	Treatment with NAC significantly reduced noise-induced threshold shifts at all frequencies (p < 0.01 compared to the exposed control group) at the frequencies of 2, 4, 6 and 8 kHz.
Fetoni et al. (2009)^27^	NAC	30 m / 120 dB / 6 kHz continuous pure tone	In the control group, a massive hair cell loss was identified in the area located 14–16 mm from the apex, the area corresponding to hair cells coding to 8–14 kHz, while the group receiving NAC treatment showed only a moderate OHC loss and normal stereocilia.
Lorito et al. (2006)^28^	NAC	4 h / 105 dB /octave band noise at 8 kHz	In TOAE analysis, only the 1500 mg/kl L-NAC group statistically differed from the control group. ABR data showed that the 1500 mg/kl L-NAC group showed threshold shifts at 8 kHz of 20 dB, 10 kHz of 15 dB, 12 kHz of 12 dB and 16 kHz of 8 dB. The study does not present the ABR results of the other groups.
Lorito et al. (2008)^29^	NAC	4 h / 105 dB / octave band noise at 8 kHz	There was a conflict between DPOAE and ABR data, which suggest that the NAC otoprotective effects should be studied in longer observation windows (>7 days), so that the full effect of ROS by-products can be assessed later.
Rhee, Chang (2021)^30^	NAC	5 h / 116 dB / Narrowband with 1/3 octave bandwidth centered at 16 kHz	No statistically significant protection was observed in functional and histological terms with NAC use.
Wu et al (2020)^31^	NAC	2 h / 100 dB /octave band noise from 8 to 16 kHz	In the evaluation of 4-HNE in OHC, treatment with NAC significantly reduced the accumulation of noise-induced ROS (p < 0.05) and significantly attenuated the AMPKα activation triggered by noise exposure (p < 0.001).
Fetoni, et al (2009)^32^	Qter	1 h / 120 dB / 6 kHz continuous tone	In the Qter-treated group exposed to noise, the shifts in mean thresholds decreased on day 7 and the values were further attenuated on day 21. The percentage of active caspase 3 cells in the area corresponding to the major damage was strongly reduced in the Qter treated cochleas. The percentage of labeled TUNEL positive nuclei was 87.7% ± 9.3% in the animals exposed to noise cochleas and 3.2 ± 4.4% in animals also exposed but treated with Qter. A significant difference in the outer hair cells loss was observed between the treated group and the control group exposed to noise.
Fetoni et al (2012)^33^	Qter	1 h / 120 dB / 10 kHz continuous tone	There was no difference between intraperitoneal and transtympanic groups, at a concentration of 40%, so the noise-induced threshold shift was not directly related to the drug treatment modality. Treatment with Qter significantly reduced OHC death at a concentration of 40% in comparison between the groups and the noise control group. In the TUNEL assay, in animals treated intraperitoneally, there was observed a decrease in the number of positive nuclei in the same area of the control group exposed to noise.
Fetoni et al (2013)^34^	Qter	1 h / 100 dB / 10 days / 10 kHz range	In animals which used Qter and were exposed to noise, there was a 60% improvement in threshold values compared to the group that did not use Qter since D11 (p < 0.0001). In DHE staining, fluorescence increased from day 1 to day 11, and the noise effects were neutralized by the use of Qter (weak fluorescence). In the Western blot analysis, Qter treatment, in the noise-exposed group, prevented initial expression on D1 and reduced immunoreactivity at the end of the treatment (D11) (p < 0.001 in the comparison of the noise × noiseQter groups on day 1 and day 11). The use of the Qter significantly reduced HC loss (p < 0.05 only in the middle gyrus in comparison with the control group) and preserved SGNs and fibers (p < 0.001 in comparison with the control in the middle gyrus and p < 0.05 in the basal gyrus).
Fetoni et al (2016)^35^	Qter	60 m / 98 dB SPL / continuous pure tone at 10 kHz / 5 consecutive days a week for 3 weeks.	The group that was exposed to noise and received Qter showed an attenuation in the decrease in DPOAE amplitude (p < 0.01 in the comparison between the previous examination and D21) and a worsening of the threshold over time, but with attenuated damage in comparison with the group that was not treated. In the D21 analysis, a statistically significant difference was observed between the groups exposed to noise in the frequencies of 6, 12, 16 and 20 kHz, with improved results in the group that received Qter (p < 0.0005). In the noise-exposed groups treated with Qter, OHC loss was significantly reduced and an increase in the number of preserved SGN was observed. There was a significant decrease in superoxide expression compared to the noise-exposed group and a reduction in lipid peroxidation in all cochlear structures compared to the noise-exposed group.
Hanci et al (2016)^36^	Resveratrol	6 h/110 dB/ 1–12 kHz	The light microscopy analysis and the transmission electron microscopy analysis showed that the group which used resveratrol presented preserved cochlear structure, hair cells and stereocilia, with an almost normal appearance compared to the group that was only exposed to noise.
Li et al (2019)^37^	Resveratrol	3,5 h/116 dB SPL/ 8 kHz (narrowband)	After exposure, the thresholds of the exposed groups, with and without drug treatment, were raised to 70.6 ± 15.8 dB (p < 0.001 vs. control group) on day 2 and recovered to approximately 51.3 ± 7.9 dB (p < 0.001 vs. control group) from day 8 to day 29, the last assessment by ABR.
Seidman et al (2003)^38^	Resveratrol	24 h/105 dB SPL/ 4500–9000 Hz	Statistically significant differences in auditory thresholds were observed in the comparison of resveratrol with the control group at the frequencies of 6 and 9 kHz at times (D0, D3, D7 and after 4 weeks). At the frequency of 12 kHz, a threshold shift was observed on D0 and D7. The mean loss of OHC in the control group was 1.3% and 0.48% in the resveratrol treatment group and no change in inner hair cells was observed.
Seidman et al (2013)^1^	Resveratrol	24 continuous hours/ broadband noise / 105 dB.	Resveratrol group showed a reduction in the intensity of COX-2 protein compared to the vehicle group (also exposed to noise). The group that received saline increased 13.6 times and the resveratrol group increased 7.3 times (p < 0.05). In the control group, there was little or no expression of COX-2.
Xiong et al. (2017)^39^	Resveratrol	1 h / NPS 120 dB / Frequency centered at 10 kHz	After 15 days of noise exposure, there was greater recovery in the noise + resveratrol group compared to the noise group at the frequencies of 4 kHz (p = 0.023) and 16 kHz (p = 0.038). There was no difference in IHC loss in the comparison between the groups exposed to noise. In the analysis of oxidative damage and 4HNE levels, a marker of oxidative stress, the use of resveratrol diet decreased 4HNE levels in OHC (p = 0.035) and GE (p = 0.016).
Minami, et al. (2007)^40^	Tempol	5 h / 120 dB / octave band noise at 4 kHz	In the comparison between the untreated group and the tempol-treated group, a reduction in the threshold shifts at 8 and 16 kHz frequencies was observed on the 10^th^ day (p < 0.05). The mean loss of OHC in the area of 5–15 mm from the cochlear apex in the untreated group was 51% and, in the group, treated with tempol was 30% (p < 0.01).
Murashita, et al. (2005)^41^	Tempol	4 h / 128 dB / pure tone at 4 kHz	A statistically significant difference was observed in the in ABR threshold shifts (p < 0.05 in the group treated with 20 mg/kg and p < 0.01 in the group treated with 30 mg/kg. No protective effect was observed after administration of 10 mg/kg (p > 0.05). Two weeks after exposure, the effect of 30 mg/kg tempol on hair cell loss (around the 3.66 mm area of the apex) was observed

### Risk of study bias

The analysis carried out by CAMARADES demonstrated the methodological quality of the studies. Uniformity of responses was observed in the topics use of the animal model used and compliance with animal welfare regulations, which were positively scored in all studies, and blinded drug therapy, which was negatively scored in all studies (Supplementary Table S1).

The SYRCLE RoB risk of bias assessment protocol revealed a high risk of bias in all eligible studies, showing inadequate randomization, unconcealed allocation, blinding of caregivers and evaluators, as shown in Fig. 1 (Supplementary Fig. S1).

### Summary of the results

The studies 1, 25, 28, 37 did not use the ABR as well as the studies 17, 21, 29, 32, which presented insufficient data for inclusion in the meta-analysis and, therefore, composed only the systematic review.

**Fig. 2:**
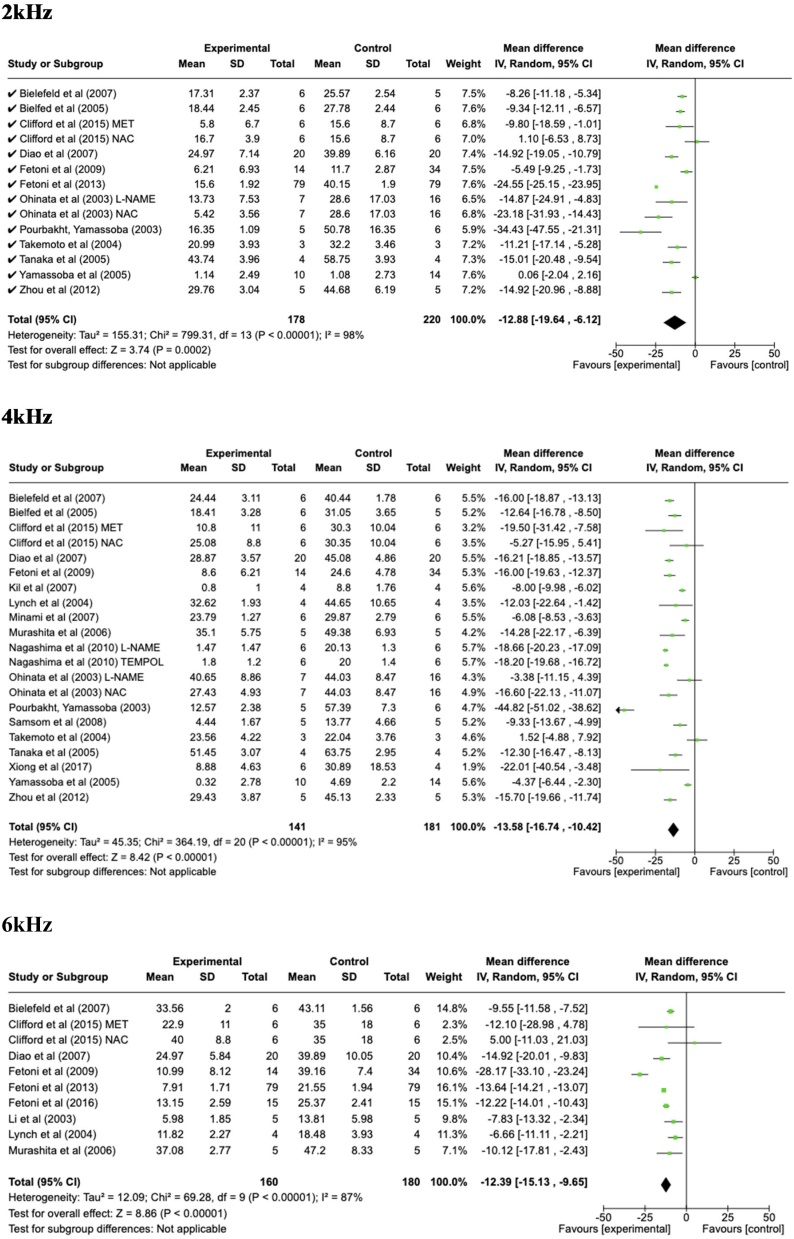

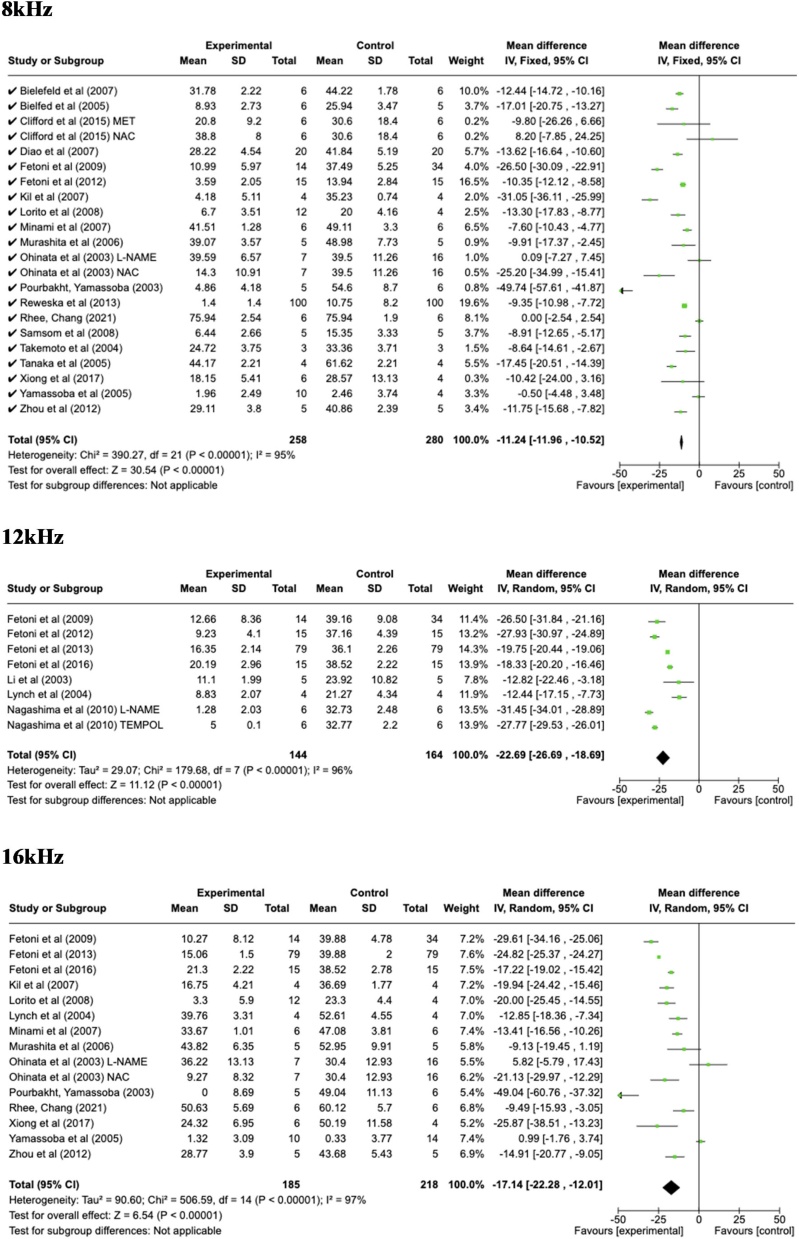

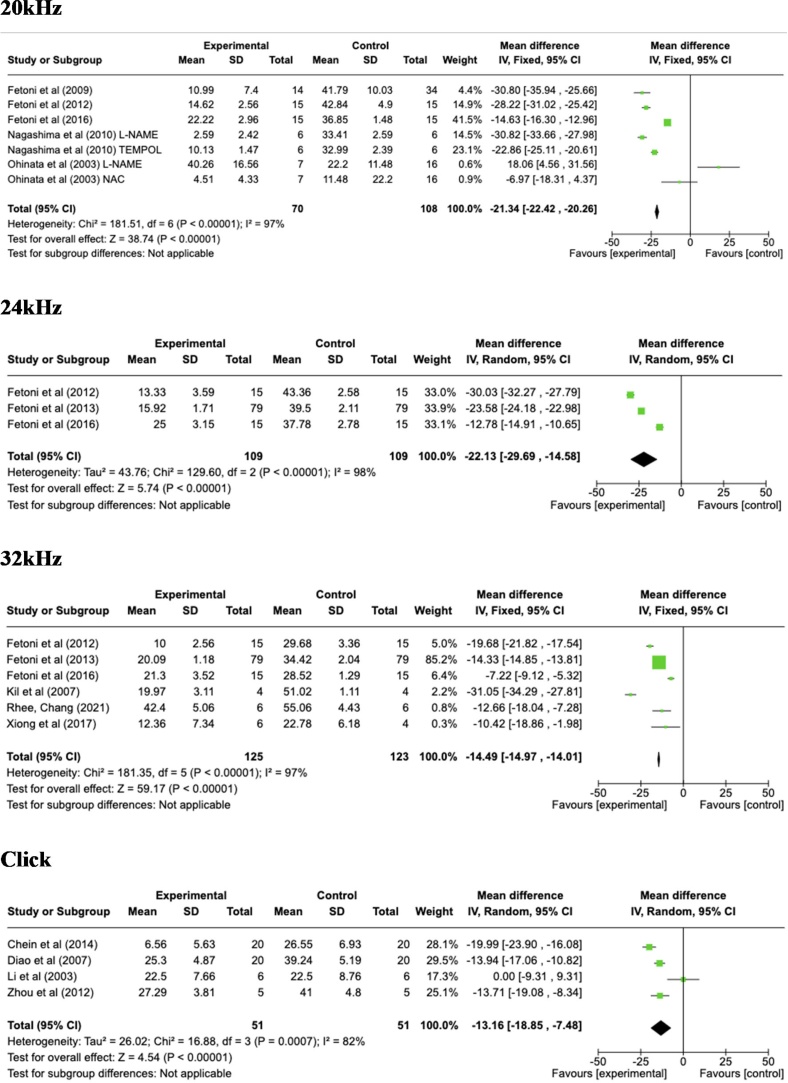
Florest Plot the analysis of frequencies from 2 to 32 kHz and click.

The data extracted for the meta-analysis were organized according to the common hearing frequencies presented by each study during the ABR test (Fig. 2).

In all frequencies evaluated, there was an otoprotective effect by the use of antioxidants in noise exposure. In 2000 Hz, the mean difference was −12.88; 95% CI −19.54 to −6.12; p = 0.0002, I^2^ = 98%; at 4000 Hz the mean difference was −13.58; 95% CI −16.74 to −10.42; p < 0.00001, I^2^ = 95%; at 6000 Hz the mean difference was −12.39; 95% CI −15.13 to −9.65; p < 0.00001, I^2^ = 87%, −12.29; 95% CI −13.00 to −11.41; p < 0.00001, I^2^ = 93%; at 8000 Hz the mean difference was −11.24; 95% CI −11.96 to −10.52; p < 0.00001, I^2^ = 95%; at 12000 Hz the mean difference was −22.69; 95% CI −26.69 to −18.69; p < 0.00001, I^2^ = 96%;, at 16000 Hz the mean difference was −17.14; 95% CI −22.77 to −12.01; p < 0.00001, I^2^ = 97%, in 20000 Hz the mean difference was −21.34; 95% CI −22.42 to −20.26; p < 0.00001, I^2^ = 97%, at 24000 Hz the mean difference was −22.13; 95% CI −29.69 to −14.58; p < 0.00001, I^2^ = 98%; at 32000 Hz the mean difference was −14.49; 95% CI −14.97 to −14.01; p < 0.00001, I^2^ = 97%; in the click, the mean difference was −13.16; 95% CI −18.85 to −7.48; p < 0.00001, I^2^ = 82%.

## Discussion

This study demonstrated the occurrence of an otoprotective effect generated by the use of antioxidants in rodents exposed to noise. Due to methodological divergences in the test execution (ABR), the different types of antioxidants, different times and types of exposures, a high heterogeneity was found in the included studies, which is described below.

It is observed, in the analysis of the systematic review studies, that there is no uniformity with regard to noise exposure. Of the 35 articles studied, exposure times ranged from 30 minutes,^22^ to 1 hour,^23^^,^^24^^,^^36^^,^^37^, ^38^, ^39^^,^^43^ 2 hours,^20^^,^^34^ 3 hours,^19^^,^^21^^,^^22^^,^^24^ 4 hours,^15^, ^16^, ^17^^,^^25^^,^^32^^,^^33^^,^^45^ 5 hours,^18^^,^^27^^,^^34^^,^^44^ 6 hours,^14^^,^^28^, ^29^, ^30^^,^^40^ 8 hours,^3^ 3.5 hours^41^ and 24 hours.^1^^,^^42^ It is also emphasized that most studies performed a single exposure to noise to demonstrate the long-term otoprotective effect, with the exception of the studies by Gao (2015)^20^ and Lynch (2004),^17^ who performed two exposures, and Fetoni (2013, 2016),^38^^,^^39^ who performed exposures for 10 days and 5 consecutive days per week, for 3 weeks.

The intensity used during noise exposure also showed high variability, with 105 dB^1^^,^^14^^,^^30^^,^^32^^,^^33^^,^^42^ and 110 dB^2^^,^^15^^,^^20^^,^^26^^,^^28^^,^^40^ being the most cited by the studies. Only one study was found which used intensity below 100 dB^39^ and three studies with intensity of 100 dB.^29^^,^^35^^,^^38^ The rest of the studies used higher intensities, such as 113 dB,^16^^,^^17^ 115 dB,^4^^,^^19^^,^^24^^,^^25^^,^^27^ 116 dB,^34^^,^^41^ 120 dB,^31^^,^^36^^,^^37^^,^^43^^,^^44^ 125 dB,^18^ 128 dB^45^ and 130 dB.^21^, ^22^, ^23^

Regarding the analysis of the noise used in its frequency range, it was observed that most studies used noise centered in a range of 4 kHz^3^^,^^4^^,^^14^^,^^15^^,^^18^^,^^19^^,^^21^^,^^22^^,^^24^^,^^25^^,^^27^^,^^29^^,^^30^^,^^44^^,^^45^; however, there were found studies that used noise centered in a range of 6 Hz,^31^^,^^34^ 8 kHz;^26^^,^^32^^,^^33^^,^^41^ 10 kHz^37^, ^38^, ^39^^,^^43^ and 16 kHz.^34^ Some studies have chosen to use a wider range of frequencies, such as 4‒16 kHz,^16^^,^^17^ 250‒4 kHz,^20^ 2.5‒3.5 kHz,^23^ 4.5‒9 kHz,^42^ 1‒12 kHz ^28^^,^^40^ and 8‒16 kHz.^35^

One of the antioxidants studied is D-methionine (d-met), which is an oral antioxidant agent, a natural component of cheese and yogurt,^2^ and was investigated by Clifford,^14^ Rewerska^3^ and Samson.^15^ The three studies were able to demonstrate over time the otoprotective effect of d-MET in animals exposed to noise. Clifford^14^ found that the use of the substance at a dose of 25 mg/day made animals recover the lowered auditory thresholds in the frequencies of 4 and 6 kHz after the third week of exposure. Rewerska^3^ demonstrated that 400 mg dose was the most efficient in preventing cochlear damage, as well as Samson^15^ who, with the same dose, found that the group which used d-met showed a significant decrease in threshold shifts in the frequencies of 4 kHz and 8 kHz.

Another antioxidant studied was Ebselen (2-phenyl-1,2-benzisoselenazole-3(2 H)-one), a selen-organic compound that minimizes glutathione peroxidase and eliminates organic hydroperoxides, which promote lipid peroxidation, with analogous removal to eliminate ROS.^18^ As a conclusion of the studies, it was observed that the longest treatment (for 14 days) was more effective than the treatment performed for only 3 days^15^ and that the higher dosage also caused a better otoprotective effect.^17^^,^^18^ The study conducted by Yamassoba^19^ was the only one which reported that animals which received Ebselen showed no change in ABR after noise exposure (3 hours at 115 dB).

Edaravone (3-methyl-1-phenyl-2-pyrazolin-5-1), cited as the first free radical scavenger used in clinical practice in Japan,^21^ which inhibits hydroxyl radicals and ameliorates iron-induced peroxidative injury^20^ was studied in noise exposure by Gao,^20^ Tanaka^21^ and Takemoto.^22^ Gao^20^ concluded that the application of Edaravone Solid Lipid Nanoparticles (SLNs) has demonstrated that it can inhibit the generation of ROS in the cochlea after noise exposure and the decrease in auditory thresholds, as measured by ABR. In the studies conducted by Tanaka et al. and Takemoto,^21^^,^^22^ the application of Edaravone was by means of an osmotic pump, implanted in the animals ears and its otoprotective effect was demonstrated by a decrease in the number of absent/defective OHCs and with a shift in the threshold at the frequency of 8 kHz, 14 days after noise exposure.^22^ The application of Edaravone, 9 hours after exposure to noise, showed a lower shift in thresholds 7 days after exposure to noise, at the frequencies of 2, 4 and 8 kHz, and a lower percentage of hair cells lost.^21^

Hydrogen, which has been described as an antioxidant reagent due to its potential to selectively reduce hydroxyl, peroxynitrite and, especially, hydroxyl radicals (most toxic ROS) to inhibit oxidative stress,^46^ was studied by and Chen,^23^ Lin^24^ and Zhou^25^). The results showed that intraperitoneal saline solution with hydrogen demonstrated efficiency in otoprotection, since the groups studied with the use of the antioxidant showed less threshold shift compared to the groups without treatment.^23^^,^^25^ The use of hydrogen water, 14 days after exposure, resulted in a statistically significant decrease in the threshold shift in the treated group only at the frequency of 4 kHz.^24^

Evidence suggests that excessive NO synthesis and ROS play an important role in NIHL, as there is increased production of NO in the cochlear perilymph after noise trauma. The NG-Nitro-L-Arginine-Methyl Ester (L-NAME), a NOS inhibitor, was studied by Diao.^4^ In their study, the group that received L-NAME (10 mg/kg) had an average NO level of 75.7 ± 15.3 mmoL/gprot, while the group that received saline solution had an average NO level of 120 ± 35 mmoL/gprot (p < 0.001). Nagashima^26^ concluded that the higher dosages (0.1 mg/kg or 1 mg/kg) showed statistically significant differences in the frequencies of 4, 12 and 20 kHz, in comparison with the control group. In disagreement with the mentioned studies, the study by Ohinata^24^ considered the L-NAME to be ineffective in comparison with the control group, with reduction in the threshold shift only at the 2 kHz frequency and an increase in the threshold shift at the 20 kHz frequency, using the same dosage as Nagashima (1 mg/kg).^26^

The study by Ohinata^27^ also involved the antioxidant N-Acetylcysteine (NAC), described as a broad-spectrum antioxidant, a consistent lipid peroxidation inhibitor and capable to act as substrate for glutathione synthesis, in addition to its own antioxidant characteristics.^27^^,^^29^ NAC was, in order of frequency, the most used substance as otoprotector in noise exposure in the studies selected for this review. In contrast to the ineffective result obtained with L-NAME, NAC attenuated OHC and IHC losses in comparison with the control group (p < 0.05), as well as in the study by Bielefeld,^29^ in which the losses of OHC were less than 10%, while the control group presented a loss of 29%–41%. The studies by Ada^27^ and Fetoni^31^ used electron microscopy to evaluate the otoprotective effect and concluded that in the NAC group there was loss of stereocilia^28^ and moderate loss of OHC,^36^ and in the control group there was a massive loss of hair cells. In the study by Wu,^34^ NAC reduced the formation of noise-induced ROS by evaluating the relative levels of 4HNE (4-Hydroxynonenal and 3-Nitrotyrosine) of OHC and significantly attenuated the activation of AMPKα (5′adenosine monophosphate-activated protein kinase), reinforcing the concept that AMPKα activation is mediated by oxidative stress. Lorito^32^^,^^33^ concluded in their studies that NAC provided different degrees of threshold reduction according to the moment of drug injection and that only the dose of 1500 mg/kg presented a statistically significant difference in the TEOAE and DPOAE analysis.

Finally, according to Bielfeld,^30^ treatment with NAC significantly reduced the noise-induced threshold shift at all frequencies evaluated. In disagreement, the studies by Clifford,^14^ with a dose of 12 mg/kg and the study by Rhee,^33^ with a dose of 100 mg/kg, concluded that the animals in the NAC group presented similar results to those of the control group, without significant hearing recovery.

Another antioxidant to show significant results was Coenzyme Q-ter, described as a multicompound that uses treated CoQ10 in association with a suitable carrier material and a bioactivator.

It is a soluble form of Coenzyme Q10, obtained by its mechanophysical activation,^39^ which is about 200 times more soluble, and its antioxidant capacity is approximately five times greater than native CoQ10. Q-ter was studied intraperitoneally, in a dose of 100 mg/kg^36^, ^37^, ^38^, ^39^ and transtympanically^37^ in doses of 20 mL e 40 mL. In the study conducted in 2012, Fetoni^37^ concluded that the 20 mls transtympanic dose showed lower threshold recovery, and the 40 mls intratympanic or intraperitoneal (100 mg/kg) doses presented similar results and significantly reduced OHC death in comparison with the control group. In 2009, Fetoni^37^ had also observed by immunohistochemistry that the percentage of active caspase 3 cells in the area corresponding to major damage was 62.0%±4.5% in noise exposed cochleas, the value was strongly reduced in cochlea treated with Qter (2.0%±1.0%), demonstrating a decrease in the signs of apoptosis. In the study developed by Fetoni in 2013,^35^ the Western blot analysis indicated that treatment with Qter reduced lipid peroxidation on exposure to noise, thus reducing oxidative imbalance and, in the study conducted by Fetoni in 2016,^39^ it was observed that the Qter administration group presented lipid peroxidative damage in all cochlear structures (spiral ganglion neurons, Corti's organ and stria vascularis) in comparison with the control group, although lower than that presented by the group exposed to noise.

Resveratrol, a polyphenol presents in many fruit and plant-based foods, widely known for its antioxidant and anti-inflammatory properties,^43^ has been studied at different doses and times of application.

In the study developed by Seidman,^1^ the analysis of cyclooxygenase-2 (COX-2) expression, which is associated with the level of cytotoxic and neurotoxic damage, was measured in animals cochlea at a single moment after 24 hours of noise exposure and demonstrated that resveratrol group showed a significant reduction in COX-2 expression compared to the vehicle-treated group (COX-2 expression increased 13.6 times in animals pretreated with saline solution compared to a 7.3 times increase in those treated with resveratrol). The study by Xiong^43^ analyzed the levels of 4-HNE, a marker of oxidative stress, and identified that animals which used resveratrol-enriched diet showed decreased levels of 4HNE in the OHCs and SNGs. In the histological evaluation performed by Seidman,^42^ it was observed that after noise exposure, the peak of OHC loss was 5.2% in the range of 7 to 9 kHz in noise exposed animals, while in the resveratrol treatment group, the peak was 2.7% in the range of 7 to 8 kHz. The light microscopy analysis demonstrated the efficiency of resveratrol because, even after noise exposure, the animals presented preserved cochlear structure and almost normal appearance compared to the group that was exposed to noise. The study by Li,^41^ which only performed ABR analysis in the click stimulus, was the only study that did not observe a difference between the results of animals that used resveratrol and those that did not. It is believed that this result was influenced by the lack of analysis of the higher frequencies, which are more affected by noise, since the studies by Xiong^43^ and Seidman^42^ found statistically significant differences in the frequencies of 4 and 16 kHz and 6 and 9 kHz, successively.

Tempol (4-hydroxy-2,2,6,6-tetramethylpiperidine-N-oxyl), a water-soluble analogue of the spin label TEMPO, which permeates biological membranes and acts as a spin trap for superoxide radicals,^44^ has also demonstrated its otoprotective potential in the studies conducted by Minami,^44^ who administered tempol in drinking water and Nagashima,^25^ Murashita,^45^ who applied tempol intraperitoneally. These last two studies^25^^,^^45^ used three different doses and both concluded that 30 mg/kg dose was the most effective in the ABR analysis, compared to the control group. The study that treated drinking water with tempol,^44^ observed a reduction in the threshold shift at the frequencies of 8 and 16 kHz (p < 0.05) in treated animals in comparison with the untreated animals 10 days after exposure to noise.

Based on the analysis of the results described, it was possible to identify in most studies the potential otoprotective effect of antioxidants use, either by protecting the threshold, improving recovery after exposure or by preserving hair cells, among others. Regardless of the form of evaluation, in most studies it was demonstrated that groups which used antioxidants presented less damage than the groups that were only exposed to noise. Meta-analysis was the performance method only for studies that utilized functional cochlear assessment through the BERA. A negative mean, in objective and functional terms, indicates an improvement in the auditory threshold in the group of animals that received the antioxidant, demonstrating the otoprotective effect of the drug in question. Regarding the mean difference value represents a clinically meaningful improvement in hearing. A reduction in the means significantly enhance auditory perception, particularly in frequencies critical for speech understanding and environmental sound detection in human been for example. Previous studies have shown that a difference of around 10–15 dB is often associated with meaningful functional improvements in hearing, especially in noisy environments or in clinical settings for hearing protection.^47^^,^^48^

However, in the methodological analysis of the studies, there are still incomplete data and divergences in methods, which impair the reliability and replicability of the studies. SYRCLE analysis revealed a high risk of bias in all eligible studies, portraying inadequate randomization, unconcealed allocation, inadequate blinding of caregivers and evaluators, and the Camarades analysis also demonstrated flaws as in 100% of the studies there is no description about blinding at the time of medication and only 6 studies stated that the results were blinded evaluated. This point is worrisome because the results of the analysis of ABR waves, the focus of the meta-analysis, can be highly influenced by the knowledge of the analyzed group in terms of identification and marking of waves to calculate latencies and interpeak. Also in relation to the ABR, great variability was observed in the criteria for wave analysis. It was clear that there is still no well-established uniform evaluation in experimental studies regarding the frequencies to be evaluated and the waves studied.

It is noteworthy that the main limitation of this meta-analysis study was the heterogeneity observed in the evaluation of all frequencies, already justified above by the methodological differences presented. It is believed that a more in-depth knowledge about the curve/dose/time/intensity of both noise and antioxidant agents may generate more robust results for a more accurate comparison, as well as the establishment of criteria for the analysis of the results, may generate a meta-analysis with less heterogeneity.

Permanent noise-induced hearing loss is one of the most common work-related diseases today. It is believed that in order to translate the results of these experimental studies in animals to humans, there is a need to create a more defined protocol on noise exposures in which noise intensity, exposure time in experimental models of rodents, thus separating the various effects of this agent on the auditory system, such as acoustic trauma, temporary threshold shifts, and permanent threshold shifts. It is believed that the results of studies with protocols using short exposures should be reviewed to prove the occurrence of permanent hearing loss, so that there are no doubts about the effects of otoprotective substances and that they are not influenced by the natural recovery of hearing thresholds caused by temporary threshold shifts.

Although the data presented in this study suggests that antioxidants possess otoprotective potential, it is acknowledged the caution required in drawing definitive conclusions regarding their efficacy and translational relevance. A key limitation of the studies reviewed is the high heterogeneity in study designs, dosages, and methodologies, which may introduce variability in the results and limit the generalizability of the findings. Additionally, several studies showed evidence of potential bias, including small sample sizes, lack of randomization, and inconsistent control conditions and variable noise exposure protocols, all of which could compromise the robustness of the results.

Given these factors, while the potential of antioxidants to protect against auditory damage is promising, further well-designed, large-scale, and rigorously controlled clinical trials are needed to validate their effectiveness and establish clear recommendations for clinical use. Even though the majority antioxidant drug is used for diet supplementation with no side effects described, the lack of powerful evidence, may limit the definitive use in clinical situation. These future studies should aim to standardize methodologies and account for confounding variables to provide more reliable and reproducible results. Until such studies are conducted, the translational application of antioxidants for hearing protection must be viewed with caution, and the results should be interpreted as preliminary rather than conclusive.

## Conclusion

This systematic review demonstrated the variable otoprotective effect with the use of antioxidants on high levels of sound pressure exposure in experimental studies in a rodent model, despite the high risk of bias. Further investigations of primary studies with methodological standardization are suggested, especially in noise exposure protocols, which are still fragile in the literature and do not contemplate the possible differences in damage to the auditory system according to the level of noise exposure.

## ORCID ID

Maria Luiza Queiroz Sampaio: 0000-0002-9754-3786

## CRediT authorship contribution statement

Gabriela Guenther Ribeiro Novanta: Participated in the study design, data collection, analysis, discussion and writing of the manuscript.

Ana Carolina Odorizzi Zica^1^: Participated in the study design, data collection, analysis, discussion, writing, and critical review of the manuscript.

Lucieny Silva Martins Serra: Participated in the study design, data collection, analysis, discussion, writing, and critical review of the manuscript.

Maria Luiza Q. Sampaio: Participated in the study design, data collection, analysis, discussion and critical review of the manuscript.

Camila Castro Correa: Participated in the data collection, discussion and critical review of the manuscript.

Andre Luiz Lopes Sampaio: Participated in the study design, discussion, writing, and critical review of the manuscript.

## Funding

This research was funded by the Federal District Research Support Foundation – FAPDF.

## Declaration of competing interest

The authors declare no have conflicts of interest.

## Footnotes

Appendix A Supplementary material related to this article can be found, in the online version, at doi:https://doi.org/10.1016/j.bjorl.2025.101696.
