# Supplementary material for: Otoprotective effect of the use of antioxidants on noise exposure in experimental studies with rodents – A systematic review with meta-analysis
Source: Braz J Otorhinolaryngol. 2025 Sep 26;92(1):101696. doi: 10.1016/j.bjorl.2025.101696 (PMC12509754; doi:10.1016/j.bjorl.2025.101696)
Supplement: Supplementary file 1 [file mmc1.docx]

**BJORL-D-24-00324_Supplementary Material**

**Supplementary Figure 1** Risk of Bias in Individual Studies assessed by SYRCLE’s RoB criteria.
