# Supplementary material for: Otoprotective effect of the use of antioxidants on noise exposure in experimental studies with rodents – A systematic review with meta-analysis
Source: Braz J Otorhinolaryngol. 2025 Sep 26;92(1):101696. doi: 10.1016/j.bjorl.2025.101696 (PMC12509754; doi:10.1016/j.bjorl.2025.101696)
Supplement: Supplementary file 2 [file mmc2.docx]

**Supplementary Table 1.** Methodological study quality assessed by adapted CAMARADES checklist.

| **Author, Year, Country** | **(1)** | **(2)** | **(3)** | **(4)** | **(5)** | **(6)** | **(7)** | **(8)** | **(9)** | **(10)** | **Total** |
| --- | --- | --- | --- | --- | --- | --- | --- | --- | --- | --- | --- |
| Clifford et al. (2015) | Y | U | U | U | U | Y | Y | Y | Y | Y | 6 |
| Rewerska, et al. (2012) | Y | Y | Y | U | U | Y | Y | Y | Y | Y | 8 |
| Samson et al. (2008) | Y | Y | U | U | Y | Y | Y | Y | Y | N | 7 |
| Kil et al. (2007) | Y | U | U | U | U | Y | Y | Y | Y | N | 5 |
| Lynch et al. (2004) | Y | U | U | U | Y | Y | Y | Y | Y | N | 6 |
| Pourbakht, A; Yamasoba, T; (2003) | Y | U | Y | U | U | Y | Y | Y | Y | N | 6 |
| Yamasoba et al. (2005) | Y | U | Y | U | Y | Y | Y | Y | Y | N | 7 |
| Gao et al. (2015) | Y | Y | U | U | U | Y | Y | Y | Y | Y | 7 |
| Tanaka et al. (2005) | Y | U | U | U | U | Y | Y | Y | Y | N | 5 |
| Takemoto et al. (2004) | Y | U | U | U | U | Y | Y | Y | Y | N | 5 |
| Chen, et al. (2014) | Y | U | Y | U | U | Y | Y | Y | Y | N | 6 |
| Lin, et al. (2011) | Y | U | Y | U | U | Y | Y | Y | Y | N | 6 |
| Zhou, et al. (2012) | Y | U | Y | U | U | Y | Y | Y | Y | N | 6 |
| Diao et al. (2007) | Y | U | U | U | U | Y | Y | Y | Y | N | 5 |
| Nagashima et al. (2014) | Y | Y | U | U | U | Y | Y | U | Y | N | 5 |
| Ohinata et al. (2002) | Y | U | U | U | U | Y | Y | Y | Y | N | 5 |
| Ada et al. (2008) | Y | Y | Y | U | U | N | Y | Y | Y | N | 6 |
| Bielefeld et al. (2005) | Y | U | U | U | U | Y | Y | Y | Y | N | 5 |
| Bielefe et al. (2007) | Y | U | U | U | U | Y | Y | Y | Y | N | 5 |
| Fetoni et al. (2009) | N | U | U | U | U | N | Y | Y | Y | N | 3 |
| Lorito et al. (2006) | N | Y | U | U | U | Y | Y | Y | Y | N | 5 |
| Lorito et al. (2008) | Y | U | U | U | U | Y | Y | Y | Y | N | 5 |
| Rhee, Chang (2021) | Y | U | U | U | U | Y | Y | Y | Y | Y | 6 |
| Wu et al. (2020) | Y | Y | U | U | Y | Y | Y | Y | Y | Y | 8 |
| Fetoni, et al. (2009) | Y | U | Y | U | U | Y | Y | Y | Y | N | 6 |
| Fetoni et al. (2012) | Y | U | Y | U | U | Y | Y | Y | Y | Y | 7 |
| Fetoni et al. (2013) | Y | Y | U | U | Y | Y | Y | Y | Y | N | 7 |
| Fetoni et al. (2016) | Y | Y | Y | U | Y | Y | Y | Y | Y | Y | 9 |
| Hanci et al. (2016) | Y | Y | Y | U | U | N | Y | Y | Y | Y | 7 |
| Li et al. (2019) | Y | Y | U | U | U | N | Y | Y | Y | Y | 6 |
| Seidman et al. (2003) | Y | U | Y | U | U | Y | Y | Y | Y | N | 6 |
| Seidman et al. (2013) | Y | U | Y | U | U | N | Y | Y | Y | Y | 6 |
| Xiong et al. (2017) | Y | U | U | U | U | Y | Y | Y | Y | Y | 6 |
| Minami, et al. (2007) | Y | U | U | U | U | Y | Y | Y | Y | N | 5 |
| Murashita, et al. (2005) | Y | Y | Y | U | U | Y | Y | Y | Y | N | 7 |

Y, Yes; N, No; U, Unclear; Total, Number of Y.

(1) Peer reviewed publication; (2) Control of temperature; (3) Random allocation to treatment or control; (4) Blinded therapy; (5) Blinded assessment of outcome; (6) Use of DPOEA/ABR to evaluate the outcomes.; (7) Animal model (ionizing radiation); (8) Sample size calculation; (9) Compliance with animal welfare regulations; (10) Statement of potential conflict of interests.
